# School-Based Suicide Risk Assessment Using eHealth for Youth: Systematic Scoping Review

**DOI:** 10.2196/29454

**Published:** 2021-09-21

**Authors:** Deinera Exner-Cortens, Elizabeth Baker, Shawna Gray, Cristina Fernandez Conde, Rocio Ramirez Rivera, Marisa Van Bavel, Elisabeth Vezina, Aleta Ambrose, Chris Pawluk, Kelly D Schwartz, Paul D Arnold

**Affiliations:** 1 Department of Psychology University of Calgary Calgary, AB Canada; 2 Werklund School of Education University of Calgary Calgary, AB Canada; 3 Child and Adolescent Addiction, Mental Health and Psychiatry Program Addiction and Mental Health – Calgary Zone Alberta Health Services Calgary, AB Canada; 4 Rocky View Schools Airdrie, AB Canada; 5 The Mathison Centre for Mental Health Research & Education Cumming School of Medicine University of Calgary Calgary, AB Canada

**Keywords:** suicide, risk assessment, youth, eHealth, school mental health, mobile phone

## Abstract

**Background:**

Suicide is a leading cause of death among youth and a prominent concern for school mental health providers. Indeed, schools play a key role in suicide prevention, including participating in risk assessments with students expressing suicidal ideation. In the context of the COVID-19 pandemic, many schools now need to offer mental health services, including suicide risk assessment, via eHealth platforms. Post pandemic, the use of eHealth risk assessments will support more accessible services for youth living in rural and remote areas. However, as the remote environment is a new context for many schools, guidance is needed on best practices for eHealth suicide risk assessment among youth.

**Objective:**

This study aims to conduct a rapid, systematic scoping review to explore promising practices for conducting school-based suicide risk assessment among youth via eHealth (ie, information technologies that allow for remote communication).

**Methods:**

This review included peer-reviewed articles and gray literature published in English between 2000 and 2020. Although we did not find studies that specifically explored promising practices for school-based suicide risk assessment among youth via eHealth platforms, we found 12 peer-reviewed articles and 23 gray literature documents that contained relevant information addressing our broader study purpose; thus, these 35 sources were included in this review.

**Results:**

We identified five key recommendation themes for school-based suicide risk assessment among youth via eHealth platforms in the 12 peer-reviewed studies. These included accessibility, consent procedures, session logistics, safety planning, and internet privacy. Specific recommendation themes from the 23 gray literature documents substantially overlapped with and enhanced three of the themes identified in the peer-reviewed literature—consent procedures, session logistics, and safety planning. In addition, based on findings from the gray literature, we expanded the *accessibility* theme to a broader theme termed *youth engagement*, which included information on accessibility and building rapport, establishing a therapeutic space, and helping youth prepare for remote sessions. Finally, a new theme was identified in the gray literature findings, specifically concerning school mental health professional boundaries. A second key difference between the gray and peer-reviewed literature was the former’s focus on issues of equity and access and how technology can reinforce existing inequalities.

**Conclusions:**

For school mental health providers in need of guidance, we believe that these six recommendation themes (ie, youth engagement, school mental health professional boundaries, consent procedures, session logistics, safety planning, and internet privacy) represent the most promising directions for school-based suicide risk assessment among youth using eHealth tools. However, suicide risk assessment among youth via eHealth platforms in school settings represents a critical research gap. On the basis of the findings of this review, we provide specific recommendations for future research, including the need to focus on the needs of diverse youth.

## Introduction

### Background

Suicide is a leading cause of death among youth in the United States and Canada [1-3]. Beyond prematurely ending the life of a young person, suicide has wide-reaching negative impacts on friends, family, and the larger community [4]. Suicide also has substantial economic costs [5]; for example, in 2010, the government of Canada estimated that suicide resulted in CAD $2.96 (US $2.35) billion in direct (eg, health care) and indirect (eg, lost productivity) costs nationally [6].

In the context of the COVID-19 pandemic, suicide risk for some youth may be elevated because of social isolation and the associated mental health impacts of the pandemic [7-9]. In addition, owing to ongoing school closures or remote learning, many youths may not have in-person contact with the school personnel who play a critical role in identifying risk for suicide and supporting students to seek help. However, school mental health providers may find themselves identifying students at risk when they connect with youth remotely, and they may be uncertain how to best support students when they are not face-to-face. Therefore, this rapid, systematic scoping review explores promising practices for conducting school-based suicide risk assessment with youth via eHealth platforms; eHealth refers to the use of information and communication technologies in health care [10]. This can include many remote technologies, such as telephone, text, Zoom, and Google Meet. Findings from this review are applicable in the immediate context of the COVID-19 pandemic and for school mental health providers who will continue to conduct eHealth risk assessments after the pandemic (eg, providers working in rural and remote settings).

### Suicide Prevention and the Role of Schools

Schools are a key suicide prevention and intervention site because of their frequent access to many youths. In the usual, non–COVID-19 context, school personnel are in regular contact with most students and thus have multiple opportunities to intervene [11-14]. Furthermore, as many youths at risk for suicide are reluctant to ask for help [15], school personnel play an important role in actively screening and referring at-risk students to appropriate community-based services (eg, mental health clinics and emergency departments). As part of this role, standardized protocols supporting school providers to assess risk effectively, cocreate safety plans with youth, and provide referral pathways to community-based interventions are essential [16,17]. Within these standardized protocols, some commonly used, standardized risk assessments that were created for use with youth in medical settings (eg, Columbia-Suicide Severity Risk Scale [C-SSRS] and Ask-Suicide Screening Questions [ASQ]) [18,19], are often used to assess suicide risk in school settings [16,17]. Conversely, a lack of standardized protocols for holistic suicide risk assessment and intervention can lead to both false positives (ie, overresponse to disclosures that do not indicate an immediate crisis) and false negatives (ie, underresponse for students in need of immediate attention) [20]. Both of these outcomes are detrimental for all stakeholders [21], including youth, families, the health care system, and schools.

Given nationwide school closures that occurred because of the COVID-19 pandemic in March 2020 (and are still ongoing in several areas), schools across the United States and Canada are now providing school mental health services, including suicide risk assessment, through eHealth platforms. In our context, partner school divisions shared that they are still attempting to implement standardized suicide risk assessment protocols remotely but do not know optimal practices for delivery (eg, building rapport and safety in a web-based environment, maintaining connections with vulnerable youth), leading to concerns about the safety and effectiveness of this process for students expressing suicide risk in these challenging times. Given the increased mental health distress some youth may experience during and following situations causing widespread loss or turmoil [22-24]—including the COVID-19 pandemic [7-9]—continued remote use of suicide risk assessment protocols is likely, and thus guidance on e-delivery is critically needed.

### Research Question and Objective

This study aims to address the following research question: What are promising practices for providing school-based suicide risk assessment to youth using eHealth? The overall objective of this review is to summarize current evidence on key recommendations for the remote implementation of suicide risk assessment protocols and apply these recommendations to the school context. To address this question and objective, we used a systematic scoping review methodology [25-28], following the PRISMA (Preferred Reporting Items for Systematic Reviews and Meta-analyses) extension for scoping reviews checklist [29]. We chose this methodology as it is appropriate for rigorous but rapid understanding of key concepts in areas not previously the focus of systematic studies. The goal is to summarize and mobilize existing research to knowledge users and decision-makers. To gather the most up-to-date information, we included both peer-reviewed and gray literature in our review. We included gray literature because we felt that substantial information on eHealth risk assessment would likely be available via professional associations and health or school authorities, and it is often better suited to rapidly respond to emerging concerns because of its closer connection to these issues in practice.

## Methods

### Peer-reviewed Literature

#### Search Strategy

The search protocol for this study was developed by the research team and reviewed by a medical research librarian and suicide prevention nonprofit organization before searches were conducted. To find relevant peer-reviewed literature for this scoping review, we searched six databases (PsycINFO, MEDLINE, Embase, CINAHL, ERIC, and Education Research Complete) on May 28, 2020. The first author conducted all the searches. The search terms were as follows: (youth OR adolescen* OR teen* OR child*) AND (risk OR suicid* OR safety OR self-harm OR self-injury OR “self-injur* behavio*”) AND (assessment* OR screen*) AND (eHealth OR telepsychology OR telehealth* OR remote* OR virtual OR web-based OR online OR mobile health OR mHealth OR telemedic* OR e-Health OR apps OR computer-based OR digital technolog* OR e-resources OR e-support* OR internet OR iphone* OR smartphone* OR teleconsult* OR tele-consult* OR tele-health* OR tele-medic* OR telemonitor* OR tele-monitor* OR telepsychiatr* OR tele-psychiatr* OR teletherap* OR tele-therap* OR tele-psychology OR virtual care OR website*). We searched the first three search strings (adolescent terms, risk terms, and assessment terms) as individual subject headings and as title or abstract search strings in each database. We searched the final search string (the technology terms) as a title search string only to increase the relevancy of returns.

#### Inclusion Criteria

**Figure 1:**
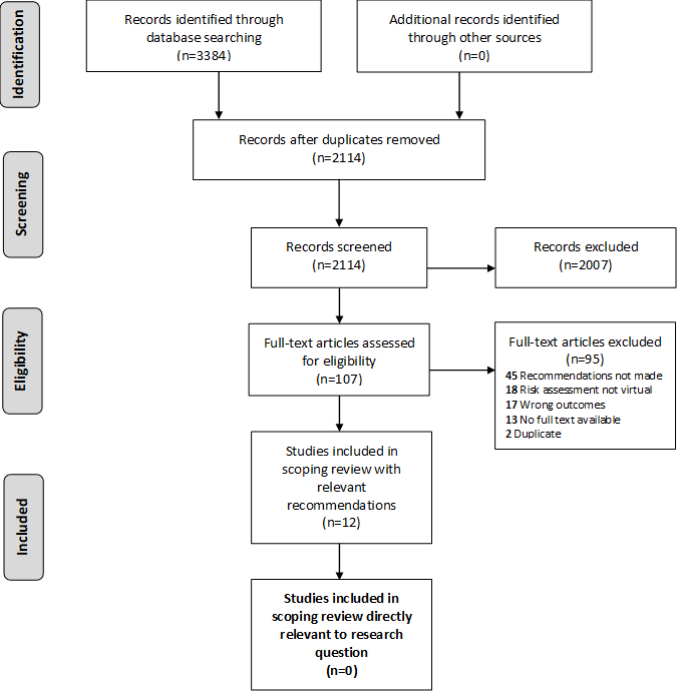
PRISMA (Preferred Reporting Items for Systematic Reviews and Meta-analyses) diagram [29,30].

Searches were restricted to peer-reviewed articles published in English in the past 20 years (ie, 2000-2020). We made this restriction as we hypothesized that most eHealth articles would be relatively recent. Indeed, a special issue on eHealth ethics was published in 2000 [10], and a Google Scholar search of the term *eHealth* indicated that the first full-text articles on this topic primarily began to appear after the year 2000. Searches were not restricted by geographic region or methodology to be broadly inclusive. To be included, articles needed to provide information relevant to completing suicide risk assessments in an eHealth (ie, remote) environment. Studies were excluded if they did not make relevant recommendations, did not focus on risk assessment, risk assessments were not completed remotely within the study, the full text was not available, or the study was a duplicate (Figure 1 [29,30]).

#### Review Procedures

The screening of the retrieved studies was completed using Covidence by a team of 5 research assistants (3 doctoral students in school and applied child psychology, 1 master of social work graduate, and 1 undergraduate psychology honors student). Research assistants reviewed the titles and abstracts of each of the 2114 potential articles in pairs (Figure 1). Each member of the pair independently reviewed each article. If the pair did not agree on an inclusion decision, they met to reach a consensus. After screening, 107 articles remained for full-text review (Figure 1). Given the expedited nature of this search, all full-text articles were reviewed by the first author. Following full-text review, 95 articles were excluded because they did not meet the inclusion criteria (Figure 1), leaving a final sample of 12 articles that provided recommendations relevant to the objective of this review (Table 1). No study specifically focused on promising practices for providing school-based suicide risk assessment to youth using eHealth. However, the 12 papers included fulfilled the overall purpose of this study (ie, relevant recommendations for remote implementation of suicide risk assessment protocols), and so they were included (Figure 1).

**Table 1:** Summary of included peer-reviewed studies (n=12).

Authors and year	Study design	Study location	Sample size	Age (years)	White, n (%)	Women, n (%)	LGBTQ-2SIA+, n (%)	Brief study description
Anderson et al [31]	Review of lessons learned	Australia	N/A^a^	12-18^b^	N/A	N/A	N/A	Shares lessons learned in the development and evaluation of a fully automated internet-based cognitive behavioral therapy (iCBT) program for youth experiencing symptoms of OCD
Arjadi et al [32]	Quantitative	Indonesia	313	24.5^c,d^	—^e^	253 (80.8)^c^	—	Presents a randomized controlled trial of the Guided Act and Feel Indonesia (GAF-ID) program, a web-based behavioral activation intervention that includes lay support
Fairchild et al [33]	Quantitative	United States	87	5-17^b^	86 (98.9)^c^	57 (65.5)^c^	—	Evaluates the outcomes of children and youth who received telemental health services within a rural emergency department
Goodday et al [34]	Review of lessons learned	United Kingdom	N/A	N/A	N/A	N/A	N/A	Reports on experiences using the True Colours remote mood monitoring system across a large number of users and settings
Haas et al [35]	Mixed methods	United States	1162	Undergraduate students	—	834 (71.8)^c^	—	Evaluates an interactive, web-based approach to encourage youth at risk of suicide to seek help
King et al [36]	Quantitative	United States	76	22.9 (5.0)^f^	54 (71.1)^c^	45 (59.2)^c^	—	Evaluates the effectiveness of a web-based intervention (*e*Bridge) for college students at risk of suicide
Navarro et al [37]	Qualitative	Australia	9	27-67^b^; 42.6 (14.3) ^f^	—	5 (55.6)^c^	—	Explores how eMental health professionals view youths’ reasons for accessing text-based counseling on the web and moderators of service delivery effectiveness
Nelson et al [38]	Literature review	N/A	N/A	N/A	N/A	N/A	N/A	Reviews the telepsychology literature (using video to deliver evaluation and/or treatment) and presents telepsychology guidance for current practice environments
Nielssen et al [39]	Quantitative	Australia	9061	18+^d^	—	—	—	Reviews procedures used to manage risk and case summaries for adults who were urgently referred for crisis intervention while using a remote screening assessment or therapy clinic (MindSpot)
Radovic et al [40]	Mixed methods	United States	96	14-26^b^	64 (67)	72 (75)	0 (0) transgender individuals	Evaluates the feasibility, acceptability, and utility of a social media website (SOVA) designed to improve mental health literacy and decrease negative health beliefs about depression or anxiety among youth with a history of depressive or anxiety symptoms
Sayal et al [41]	Mixed methods	United Kingdom	22	16-30^b^	21 (95)^c^	17 (77)^c^	—	Reviews the feasibility of a randomized controlled trial of a remotely delivered problem-solving cognitive behavior therapy for youth with repeat self-harm and depression called e-DASH^g^
Thomas et al [42]	Quantitative	United States	494	1-19^b^; 13.2 (2.6)^c,f^	360 (72.9)^c^	297 (60.1)^c^	—	Evaluates a telepsychiatry program at a geographically dispersed pediatric emergency department

^a^N/A: not applicable.

^b^Age range.

^c^Hand calculated from the information in the article.

^d^Sample age.

^e^Not available.

^f^Mean age (SD).

^g^e-DASH: Electronic–Depression and Self-Harm.

#### Data Abstraction

Data from the 12 articles included were extracted using a standardized data charting template created for this study, based on the recommendations of Tricco et al [29] and Levac et al [27]. The standardized data charting template collected information on the study’s source of funding, design, sample size, age, demographics, setting or location, data analyses, and relevant information about eHealth suicide risk assessment among youth. Abstractions were completed in pairs by a team of research assistants. Each member of the pair independently reviewed their assigned articles, and the pair then met to reach a consensus on the final abstraction. Abstractions were reviewed by the first and second authors. We did not assess data quality, as this is outside the parameters of scoping reviews [29].

To organize information extracted using the standardized data charting template into overall recommendation themes, the first author inductively applied codes to extract data using Dedoose, a web-based mixed methods data analysis software. Codes were then reviewed and revised by the second author, and the 2 authors met to arrive at a consensus on any discrepancies. From this coding, five recommendation themes emerged from 12 peer-reviewed articles.

### Gray Literature

To supplement our peer-reviewed article search, we also included key websites (ie, professional websites focused on school mental health, suicide prevention, or youth mental health) as a gray literature source. We chose to focus on websites in this part of the search as we felt these would have the most up-to-date information on remote suicide risk assessment among youth in the context of the COVID-19 pandemic. Websites for inclusion were identified by the research team and a suicide prevention nonprofit organization. These websites are not geographically restricted. Between May 28 and June 19, 2020, we reviewed 17 websites for information relevant to eHealth suicide risk assessment among youth (Multimedia Appendix 1).

Each website was thoroughly reviewed by a research assistant, and potentially relevant documents or information were saved to a shared folder. These potentially relevant documents or information were then reviewed by a separate pair of research assistants (ie, not including the research assistant that originally pulled documents from the website) to determine if they met the study inclusion criteria. From these 17 websites, we identified 23 gray literature documents that met inclusion criteria. We note that we found three different offerings of similar webinar content about suicide risk assessment via eHealth during this search. In addition to the Suicide Prevention Resource Center webinar titled *Treating Suicidal Patients During COVID-19: Best Practices and Telehealth* [43], this content was also offered as part of the Mental Health Technology Transfer Center Network’s Clinical Innovations in Telehealth Learning Series [44] and as part of a School-Based Health Alliance/National Center for School Mental Health webinar [45]. Therefore, only the Suicide Prevention Resource Center [43] webinar was included in the review (Multimedia Appendix 2 [31-43,46-67]).

Once the 23 relevant documents were found, the pair of research assistants then abstracted information using the same procedure employed for peer-reviewed articles. The standardized data charting template for gray literature included the title of the relevant page on the website and a summary of relevant information about remote suicide risk assessment among youth. The abstracted information was then reviewed and themed by the first and second authors using the same procedure as for peer-reviewed articles (see above). Finally, themes from the peer-reviewed literature were compared, contrasted, and integrated with themes from the gray literature by the first and second authors. From this process, we ended up with six total recommendations (four that were supported by information from both the peer-reviewed and gray literature, one supported by information in peer-reviewed literature only, and one supported by information in gray literature only; see the *Results* section for more information).

## Results

### Peer-reviewed Literature

#### Description of the Included Articles

We did not find any study that specifically addressed our research question (ie, promising practices for conducting school-based suicide risk assessment with youth via eHealth). However, we found 12 articles that provided information relevant to the overall study purpose and that we felt could inform future research and practice on school-based suicide risk assessment with youth via eHealth within the parameters of a scoping review (Table 1). The included articles used samples from the United States (n=5), Australia (n=3), the United Kingdom (n=2), and Indonesia (n=1; Table 1). All articles were published between 2008 and 2020, with most (10/12, 83%) published since 2015. The most common study design was quantitative (5/12, 42% of the included articles). For nonreview articles, most samples comprised youth aged between 12 and 25 years (Table 1). We also included one qualitative study with a sample consisting of eMental health professionals [36] and one study that described risk assessment outcomes for an eMental health clinic for adults, as the recommendations were highly relevant to our study [38]. The sample size of the included articles ranged from 9 to 9061 participants (Table 1).

Although youth, in general, experience an elevated risk of suicide [68], youth who experience marginalization because of certain aspects of their identity (eg, Indigenous youth, newcomer and refugee youth, immigrant youth, lesbian, gay, bisexual, trans, queer/questioning, two-spirit, intersex, asexual (LGBTQ2SIA+) youth, and youth with disabilities [69-71]) are at disproportionate risk. Owing to stereotypical gender role norms that discourage help-seeking, male youths are also at heightened risk [68,71]. Thus, we specifically explored whether the reviewed studies considered race or ethnicity, gender, and/or LGBTQ2SIA+ identity in their design (Table 1; none of the studies provided information on disability or citizenship status). Of the relevant studies (ie, original empirical studies, n=9), we found that 89% (8/9) reported on participant cisgender, 56% (5/9) reported on participant race or ethnicity, and 11% (1/9) reported on LGBTQ2SIA+ identity (by stating there were 0 transgender individuals in their sample [40]). Where cisgender and race or ethnicity were reported, samples were primarily female (range 55.6%-81%; median 68.9%) and White (range 67%-98.5%; median 72.9%). Thus, relevant recommendation themes should be interpreted with caution, as they may primarily pertain to White, cisgender female and likely heterosexual youth.

#### Relevant Recommendation Themes

By coding abstracted data, we identified five overall recommendation themes in the 12 peer-reviewed articles: (1) accessibility, (2) consent procedures, (3) session logistics, (4) safety planning, and (5) internet privacy. A summary of recommendation themes is provided in Textbox 1, and full information on themes and underlying recommendations is provided in Multimedia Appendix 2.

**Summary of recommendations from peer-reviewed and gray literature.**

**Promising practice and relevant recommendations**

- Youth engagement: accessibility, building rapport, establishing a therapeutic space, and helping youth prepare for remote sessions (recommendation from peer-reviewed and gray literature)
  Choose a mode of technology that meets the youth’s needs or preferences (eg, consider internet access, if they are comfortable with video, and minutes available on phone plans)
  Test technology before sessions and ensure devices are fully charged
  Brainstorm ways to increase youth’s sense of privacy (eg, having a codeword if someone is nearby and picking a time of day when the house is quieter)
  Discuss what virtual sessions will look like and ask youth what they need from the virtual relationship (eg, how can you make them feel safe and secure)
  Make sure youth can see and hear you clearly the entire time and that they know you are the only one in your room
  Set up the session to promote comfort and minimize distractions (remove personal items from your room, make sure your room is not distracting, let youth know they can be informal and use a background or emojis, and encourage the youth to be in a quiet part of their house)
  Use your facial expressions to convey warmth and enthusiasm, and give youth space to speak
  Keep youth engaged through various methods (eg, using screen sharing or playing a game)
  Consider providing youth with session transcripts to help them remember information and strategies to use in daily life
- School mental health professional boundaries (recommendation from gray literature only)
  Make sure youth and caregivers know when you are and are not available and who to contact when you are not available
  Use an institutional (not personal) device, and have a clear schedule for when you meet with youth
- Consent procedures (recommendation from peer-reviewed and gray literature)
  Describe what the youth should expect to happen during sessions, what technology will be used (eg, if it is recorded), and the risks and benefits of eHealth
  Obtain contact information for multiple caregivers (if possible) in case one is not available when you attempt to reach them
  Provide information on who the youth or caregiver should contact in an emergency
  Describe what will happen if the youth is determined to be at an immediate safety risk
  Detail the plan for what happens if the connection is lost during a session (eg, backup phone number)
  Obtain caregiver consent and youth assent
- Session logistics (recommendation from peer-reviewed and gray literature)
  Have a plan for what to do if you get disconnected or need to get immediate support to the youth (eg, call a caregiver and have emergency services arrive)
  Practice using the technology before the call starts, and make sure you are competent with the platform
  Make sure the technology meets relevant privacy requirements
  Verify the youth’s identity at the start of the session (if you have not met them before or if you cannot see them) and confirm the youth’s physical location
  Monitor how the youth is feeling throughout the session
  Document when the assessment started and ended, what platform you used, if you had any technical difficulties, specific topics covered, and any other notes
  End the session by asking what could be improved for next time
  If a youth misses the session, check in to see how they are and what you can do to make the sessions easier or more comfortable for them to attend
- Safety planning (recommendation from peer-reviewed and gray literature)
  Follow the same basic steps as in-person risk assessments (eg, completing a safety plan and having information for in-person resources and emergency services ready before the session in case needed)
  If the risk is not immediate, develop a safety plan and send it to the youth and their caregiver (eg, text it to them, email, or have them take a screenshot) and include contact information for 24-hour resources
  Consider using ongoing screening data to continually monitor risk (eg, agree to check in every day, week, or biweekly [depending on risk] using web-based assessments), and provide youth with feedback on risk indicators
  Inform caregivers if suicide risk issues arise and provide clear guidelines on how to manage risk and seek appropriate help
- Internet privacy (recommendation from peer-reviewed literature only)
  Send virtual session invitations via a secure and encrypted email
  Give each youth a unique, nonidentifiable username and password
  Store youth information (eg, email addresses) in an encrypted computer system and use encrypted point-to-point technologies when videoconferencing
  Ensure the virtual session hosting platform is compliant with relevant health privacy laws in your area (eg, Health Insurance Portability and Accountability Act)

#### Accessibility

Two articles discussed recommendations relevant to the accessibility of eMental health services, which also seemed potentially relevant for using eHealth for youth suicide risk assessment within school settings. Both articles were original empirical studies. One original study from Indonesia was conducted with youth whose mean age was 24.5 years [32], and the other from Australia was a qualitative study with service providers [37]. The samples were primarily female (median 68.3%). In their study, Arjadi et al [32] discussed that there are important contextual factors concerning accessibility to consider when working in the eHealth environment, such as ensuring that service delivery is accessible for individuals living in poverty, in rural areas, and/or for those with restricted internet access (Multimedia Appendix 2).

#### Consent Procedures

Two articles discussed recommendations relevant to consent procedures in the eHealth environment. One of these articles was an original empirical study from the United Kingdom conducted with individuals aged between 16 and 30 years (21/22, 95% White and 17/22, 77% female [41]), and the other was a review article [38]. Recommendations included ensuring that the provider had the name and contact information of the primary caregiver (and, given the potential need to contact someone quickly, the names and contact information for several other supportive adults before starting the session, in case an urgent suicide risk emerged; Multimedia Appendix 2). It is also important that the youth or caregiver knows who they should contact in case of a crisis, especially when the school-based provider is not available, and that the consent form describes the risks and benefits of eHealth services (Multimedia Appendix 2).

However, one of our expert reviewers (a school psychologist) noted that the consent procedures described by these studies did not fully apply to the school context (particularly the typical school requirement for caregiver involvement when suicide risk is present, whether or not this is something desired by the youth). In a typical face-to-face school setting, providers are able to conduct a suicide risk assessment without caregiver consent because there are adults in the school who will monitor the youth for safety throughout the process (ie, once a disclosure has been made, youth are never left alone). At the end of the risk assessment process, the school provider then contacts the youth’s caregiver so that the caregiver can continue supervision as part of the safety plan [17]. However, in the eHealth environment, school providers generally need to notify the caregiver *before* beginning the risk assessment process to ensure the youth’s immediate safety (ie, a caregiver notification is typically sent during the initial assessment in the eHealth environment and not during the safety planning process as in the face-to-face environment). If it is not safe to contact the caregiver, and the need is urgent, emergency services may need to be called to bring the youth to a setting for the assessment where there is supervision. Given these differences in the school (compared with the general community) environment, the existing literature on consent for eHealth suicide risk assessment does not completely align with school-based needs. Thus, research specific to conducting eHealth suicide risk assessment with youth in the school environment is critically needed.

#### Session Logistics

Five studies discussed recommendations relevant to eHealth session logistics. Four of these papers were original empirical studies from the United States, Indonesia, and Australia [32,35,39,40], and one was a review article [38]. Where information was reported, the original studies were conducted primarily with older, predominately female youth and/or adults. For the studies conducted in the United States and Australia, samples were primarily White. From their experience working with more than 9000 adults in an eMental health setting in Australia, Nielssen et al [39] concluded that (for adults) web-based suicide risk assessments should follow the same basic steps as in-person risk assessments and include specific protocols and procedures. However, given nuances in the eHealth environment (eg, nonverbal clues, how to communicate the safety plan to youth and caregivers), service providers should receive specific training on how to conduct suicide risk assessments via eHealth (Multimedia Appendix 2). Providers should also have a backup plan in case the youth is in crisis and internet and/or technology issues occur, and they should go over this plan with the youth (and caregivers) at the beginning of the session (Multimedia Appendix 2). Finally, it is important that providers understand the relevant professional requirements for providing mental health services remotely to youth at risk of suicide.

#### Safety Planning

Nine studies discussed recommendations relevant to eHealth safety planning. Six of these papers were original empirical studies from Australia, the United Kingdom, the United States, and Indonesia [32,33,36,39,41,42], and three were review papers [31,34,38]. Where information was reported, the original studies were conducted primarily with older, predominately female youth and/or adults. For the studies conducted in the United States, the United Kingdom, and Australia, samples were primarily White (median 84.2%). A relevant recommendation emerging from these studies is the potential use of screening data and personalized feedback to remotely monitor risk and increase youth engagement, respectively (Multimedia Appendix 2). Specifically, as school mental health providers may not interact with students daily, these screening data can help providers monitor emerging risks in the remote environment. Related to this, Nelson et al [38] suggested that more check-ins may be required when using a web-based format compared with the in-person environment, especially for youth who are more isolated (eg, youth living in rural settings). Finally, it is important to provide clear guidelines to caregivers on how to manage risk and seek appropriate help (Multimedia Appendix 2).

#### Internet Privacy

Four studies discussed recommendations relevant to internet privacy when providing eMental health services. Three of these papers were original empirical studies from the United States and Indonesia [32,35,42], and one was a review paper that discussed internet privacy issues [38]. Where information was reported, original study samples primarily comprised older, predominately female youth, and samples from the United States were predominately White. Recommendations included using an encrypted email to send session invitations and ensuring that the remote session hosting platform is compliant with relevant health privacy laws (Multimedia Appendix 2). Storage of youth information (eg, email addresses and cell phone numbers) is also an important privacy consideration (Multimedia Appendix 2). Reviewing the telepsychology literature, Nelson et al [38] also recommend asking youth who else is in the room, and whether they are comfortable with those people there or whether those people’s presence complies with relevant health privacy laws and institutional requirements. Who is in the room should also be considered by the service provider to ensure that the risk assessment is conducted privately and confidentially.

### Gray Literature

Overall, there was more specific and detailed information in the gray literature (ie, documents from relevant websites) on using eMental health with youth, both generally and for conducting suicide risk assessments. From the 17 websites (Multimedia Appendix 1), we extracted relevant information from 23 documents. In general, these 23 resources highlighted that during the COVID-19 pandemic, it is especially important to assess youth at risk of suicide on an ongoing and regular basis, given the stressful changes many youths are experiencing [46]. A summary of recommendation themes from the peer-reviewed and gray literature is provided in Textbox 1, and full information on themes and underlying recommendations is provided in Multimedia Appendix 2.

Specific recommendations from the gray literature substantially overlapped with and enhanced three of the themes identified from the peer-reviewed literature—consent procedures, session logistics, and safety planning; there were no additional recommendations in the gray literature about internet privacy (Multimedia Appendix 2). In addition, per findings from the gray literature, we expanded the aforementioned *accessibility* theme to a broader theme termed *youth engagement*, which included information on accessibility, building rapport, establishing a therapeutic space, and helping youth prepare for remote sessions (Multimedia Appendix 2). Finally, one new theme was identified in the gray literature findings, specifically around school mental health professional boundaries. This theme emphasized the importance of establishing when the school mental health provider would and would not be available in the remote environment and advised arranging coverage periods when possible (Multimedia Appendix 2).

Besides the additional detail provided for specific recommendation themes, a second key difference between the gray and peer-reviewed literature was the former’s focus on issues of equity and access and how technology can reinforce existing inequalities [72]. A number of resources specifically detailed that providers must consider youth’s ability to use different remote technologies and ensure that care is accessible (eg, considering the internet, data and/or phone minute restrictions; Multimedia Appendix 2). For example, in a webinar from the Mental Health Technology Transfer Network [73], presenters highlighted that each youth should be evaluated based on their individual, communal, and national culture (eg, how different cultures demonstrate pain or distress). This study also highlighted that certain perceived accents might be difficult for some providers or youth to understand, and it is thus key that providers recognize when someone may have difficulties understanding them or when they may have difficulties understanding someone else [73]. Regarding language diversity, efforts should be made to provide services in the language in which the youth are most comfortable [73]. Efforts should also be made to increase accessibility for any resources provided (eg, closed captions and sign language [73]). Overall, it is critical for school mental health providers to explore “intersections of culture, sociodemographics, geography, and technology” when using mental health services—including eHealth suicide risk assessment—with youth [74].

## Discussion

### Key Findings

In this systematic scoping review, we found six key recommendation themes across the included peer-reviewed and gray literature sources: (1) youth engagement (accessibility, building rapport, establishing a therapeutic space, and helping youth prepare for remote sessions), (2) school mental health professional boundaries, (3) consent procedures, (4) session logistics, (5) safety planning, and (6) internet privacy. We believe these recommendation themes (general summary of recommendations is outlined in Textbox 1; a detailed summary of recommendations is outlined in Multimedia Appendix 2) will be helpful to school mental health providers as they continue to conduct suicide risk assessments with youth via eHealth during the COVID-19 pandemic and beyond. However, as these recommendations are generally from research conducted outside the school setting, they should be applied with caution (eg, recommendations about consent procedures). Research specific to conducting eHealth suicide risk assessment with youth in the school environment is critically needed.

In our comprehensive search of the peer-reviewed literature, we found no peer-reviewed articles that specifically described promising practices for conducting suicide risk assessments (school-based or otherwise) with youth via eHealth. Thus, this represents a major gap in the literature. Although suicide risk assessments have been developed for use with youth in medical settings [18,19], to our knowledge, none of these screeners have been specifically adapted for or tested for use in the school-based context, and, even when they are used within the contexts for which they were designed, suicide risk screeners do not have strong diagnostic accuracy [75,76]. Future studies need to (1) focus on increasing the diagnostic accuracy of suicide risk screeners for youth, (2) adapt and test these screeners within the school-based context, and (3) specifically explore school-based versus community or medical suicide risk assessments for youth delivered via eHealth because of the different requirements in these settings (eg, around confidentiality and liability). The 12 peer-reviewed papers that provided relevant information were also primarily focused on White, cisgender female, and presumably heterosexual youth. As groups at disproportionate risk for suicide include cisgender male youth, Indigenous youth, immigrant, newcomer and refugee youth, LGBTQ2SIA+ youth, and youth with disabilities [71,77], this represents a further gap in the literature. Therefore, new research in this area should focus on expanding the diversity of youth participants. In addition to including diverse groups of youth, it is also critical that future research on this topic centers youth voices and experiences [40].

Although we found very limited peer-reviewed literature, we found many gray literature documents that provided specific information on conducting remote suicide risk assessments with youth. Many of the resulting recommendations overlapped and expanded upon the limited information available in the peer-reviewed literature. Thus, although more research on promising practices for conducting suicide risk assessment with youth via eHealth in the school setting is required, as school mental health providers are in need of immediate guidance in the face of the COVID-19 pandemic, we feel the key recommendation themes extracted from peer-reviewed and gray literature sources in this review represent a set of six promising practices for current implementation. However, research evidence is needed for these recommendations before widespread adoption and to support the development of effective school-based suicide risk assessments for youth.

The lack of peer-reviewed studies in this review reflects the state of the e-suicide prevention literature more broadly. Specifically, in their systematic review of mobile or web-based suicide prevention literature published between 2000 and 2015, Perry et al [78] found only one study that met their inclusion criteria (studies with youth aged 12-25 years; included suicidality as a primary outcome using any study design; and, published in English in a peer-reviewed journal). Given the increased access that e-suicide interventions (including risk assessment) offer youth in the context of COVID-19, and post–COVID-19 for rural and remote youth, it is critical that future research address this gap. Recommendations from gray literature sources provide rich information on which to base this work.

In addition, although we did locate a number of studies that examined eMental health interventions generally for youth, almost none of them described their risk assessment procedures for participants experiencing suicidal ideation; thus, these articles were excluded from this review. As such, we recommend that future eMental health intervention research be explicit about describing procedures for how youth suicide risk can be assessed remotely.

In the context of the COVID-19 pandemic, new research is emerging rapidly, including on the topic of suicide prevention. For example, a recent article by Szlyk, Berk, Peralta, and Miranda [79] (published after we conducted our peer-reviewed literature search at the end of May 2020) explored the implications of COVID-19 for adolescent suicide prevention. In this paper, Szlyk et al [79] recommend several evidence-based strategies to address mental health needs and decrease the risk of suicide among adolescents during the COVID-19 pandemic. First, they recommend restricting access to potential means of suicide (eg, firearms, medication, and knives). Second, for adolescents with a history of suicide attempts, ideation, or self-harm, they recommend that caregivers consider limiting their time spent alone. Third, caregivers and school mental health providers can collaboratively monitor social media use with the youth and help set healthy limits around internet use. They also suggest that school mental health providers have frequent check-ins with adolescents and create a safe space where they can have open discussions about their feelings. Fourth and finally, Szlyk et al [79] recommend taking any discussion of self-harm or suicide seriously; for school mental health providers, this means continuing to conduct risk assessments remotely, and contacting emergency services when indicated per the assessment. These four recommendations align with many of the recommendations of our review.

Furthermore, research is also emerging on advanced methods to improve accurate prediction of suicide attempts, specifically, complex algorithms that can consider a large number of risk factors simultaneously (vs more traditional methods of regression analyses with several predictors). These complex algorithms can be implemented with machine learning, a type of artificial intelligence that learns patterns from data and then applies that knowledge automatically to improve risk prediction. Researchers have recently used this technique to analyze thousands of complex health records to predict which patients are at risk of attempting suicide, leading to a more clinically accurate risk prediction [80,81]. As this technique does not require face-to-face screening, it would be particularly useful in the context of eHealth. Thus, we recommend future research that considers how we can leverage this technology to further enhance suicide risk assessment and intervention efforts with youth.

### Limitations

Our study has several limitations. First, many of the specific recommendations made in Multimedia Appendix 2 came from the gray literature, which is generally not independently or peer-reviewed; thus, these recommendations require testing with youth. Second, although we considered any remote approach to fall within our definition of eHealth, most of the recommendations we found were for mediums that included a voice component. As text-based interactions are a different context that likely require a different skill set, the recommendations from our review may not apply to text-based approaches. Finally, and as noted above, almost none of the literature we reviewed (peer-reviewed or gray) was specific to the school setting, and thus recommendations should be applied with caution by school mental health providers.

### Conclusions

From this rapid, systematic scoping review, we conclude that promising practices for conducting suicide risk assessments with youth via eHealth in school settings represents a critical research gap. Future research with diverse groups of youth is required to address this gap. However, for school mental health providers searching for immediate guidance, we feel the recommendations in this review represent the most current promising practices for suicide risk assessment with youth via eHealth until additional research is available.

## Appendix

Multimedia Appendix 1 Websites reviewed for relevant gray literature.

Multimedia Appendix 2 Detailed relevant recommendations from peer-reviewed and gray literature.

## Abbreviations

LGBTQ2SIA+: lesbian, gay, bisexual, trans, queer/questioning, two-spirit, intersex, asexual
PRISMA: Preferred Reporting Items for Systematic Reviews and Meta-analyses
