# Supplementary material for: School-Based Suicide Risk Assessment Using eHealth for Youth: Systematic Scoping Review
Source: JMIR Ment Health. 2021 Sep 21;8(9):e29454. doi: 10.2196/29454 (PMC8493464; doi:10.2196/29454)
Supplement: Multimedia Appendix 1 [file mental_v8i9e29454_app1.docx]

**Multimedia Appendix 1.** Websites Reviewed for Relevant Grey Literature.

| **Website** |
| --- |
| https://www.apa.org/advocacy/suicide-prevention/ |
| https://www.canada.ca/en/public-health/services/publications/healthy-living/suicide-prevention-framework.html |
| https://www.csmh.uwo.ca |
| https://www.cymh.ca/en/index.aspx |
| https://www.integration.samhsa.gov/clinical-practice/suicide-prevention |
| https://www.mentalhealthcommission.ca/English/what-we-do/suicide-prevention |
| https://mhttcnetwork.org |
| http://www.schoolmentalhealth.org |
| https://smho-smso.ca |
| http://www.sprc.org |
| https://www.suicideinfo.ca |
| https://suicideprevention.ca |
| https://suicidepreventionhub.org.au |
| https://suicidepreventionresearch.ca |
| http://teenmentalhealth.org/ |
| http://www.togethertolive.ca/prevention-tools-and-resources |
| https://zerosuicide.edc.org |
