# Supplementary material for: School-Based Suicide Risk Assessment Using eHealth for Youth: Systematic Scoping Review
Source: JMIR Ment Health. 2021 Sep 21;8(9):e29454. doi: 10.2196/29454 (PMC8493464; doi:10.2196/29454)
Supplement: Multimedia Appendix 2 [file mental_v8i9e29454_app2.docx]

**Multimedia Appendix 2***.* Detailed Relevant Recommendations from Peer-Reviewed and Grey Literature.

| Promising Practice | Relevant Recommendations: Peer-Reviewed Literature | Relevant Recommendations: Grey Literature |
| --- | --- | --- |
| **1. Youth engagement: Accessibility, building rapport, establishing a therapeutic space and helping youth prepare for**  **remote sessions** | - Ensure sessions are accessible for youth with restricted internet access (e.g., youth living in poverty, rural/remote youth; Arjadi et al., 2018) - Consider providing youth with session transcripts to help them remember information and strategies to use in daily life, if aligned with privacy requirements (Navarro et al., 2020) | - Make sure youth can see and hear you clearly (CYMH & School Mental Health Ontario [SMH], 2020; Reinert, 2020; Van Dyk et al., 2020) , and that your screen is big enough to see the youth’s face (CYMH & SMH, 2020)   - Both auditory and visual information are key to providing good virtual care (CYMH & SMH, 2020), so it is ideal to have both sources of information if possible given the youth context   - It can be harder to pick-up on non-verbal cues in the virtual environment, so pay special attention to facial cues (CYMH & SMH, 2020). Also pay attention to youth’s tone of voice, use of negative language and atypical speech patterns (Mental Health Technology Transfer Centre Network [MHTTC Network], n.d.). - Stay on screen the entire time and maintain eye contact (American Psychological Association [APA], 2020b; Cox, 2020). Make sure youth can clearly see your face throughout the session (Reinert, 2020; Van Dyk et al., 2020). - Discuss how to increase youth privacy (e.g., picking a time of day for the session that is quieter in the house, having a code word if someone is nearby, using chat if they can’t speak privately, wearing headphones, password-protected sessions) (Cox, 2020; CYMH & SMH, 2020; National Association of School Psychologists [NASP], 2020a; Reinert, 2020; ZERO Suicide Institute, n.d.b) - Choose a mode of technology for sessions that meets youth needs/preferences (APA, 2020b; Cox, 2020; NASP, 2020a; NASP, 2020b; ZERO Suicide Institute, n.d.b)   - Equity and access issues are critical to consider (American School Counselor Association [ASCA], n.d.; CYMH & SMH, 2020). This can include using the phone/texting when internet is not an option (CYMH & SMH, 2020), and checking with youth how many phone minutes they have to talk (MHTTC Network, n.d.). - Set-up your room to promote youth comfort (e.g., remove personal items, minimize distractions, use a headset so your voice is clear, ensure you are well-lit, set-up your computer so you can maintain good eye contact) (Alvord, Baker & Associates, LLC, n.d.; APA, 2020b; CYMH & SMH, 2020; Van Dyk et al., 2020). Let youth know they can be informal and use a background or emojis if that makes them more comfortable (Van Dyk et al., 2020). - Discuss what virtual/remote sessions will look like (e.g., security concerns, whether the session will be recorded) (Alvord, Baker & Associates, LLC, n.d; Van Dyk et al., 2020). Reassure youth at start of the session that you are in a private space. (Cox, 2020; CYMH & SMH, 2020; Reinert, 2020). Let the youth see your whole office (CYMH & SMH, 2020; Van Dyk et al., 2020). - Keep youth engaged (e.g., use screen sharing, play a game together, have youth share a photo, show them things in your environment like art or toys) (CYMH & SMH, 2020; Lowenstein, n.d.; Van Dyk et al., 2020) - Give the youth space to speak, since this can be more difficult in the remote environment (Van Dyk et al., 2020). Let youth know they can interrupt you at any time if they need to tell you something (CYMH & SMH, 2020). - Convey warmth and enthusiasm through your facial expressions and tone (Lowenstein, n.d.; Reinert, 2020) - Ask youth what they need from your virtual relationship, and how you can make them feel safe and secure (CYMH & SMH, 2020) - Help youth find a quiet, private place for your sessions where they will have minimal distractions (American Psychiatric Association, 2020; MHTTC Network, n.d.). Involve caregivers to make sure the space is private for the duration of your session (Cox, 2020). - Encourage youth to test out technology before using (ZERO Suicide Institute, n.d.a) - Remind youth to make sure their phone (or other device, such as a laptop) is fully charged before the session (Cox, 2020; MHTTC Network, n.d) - Suggest turning off smart devices to increase privacy (e.g., Alexa, Google Home) (Cox, 2020) - Encourage youth to write down what they want to talk about before session, and to bring a paper and pen to the session to take notes (American Psychiatric Association, 2020; MHTTC Network, n.d.) |
| **2. School mental health professional boundaries** | - No specific recommendations provided | - Make sure youth and caregivers know when you are and aren’t available, and who to contact when you are not available (ASCA, n.d.; CYMH & SMH, 2020; NASP, 2020a; NASP, 2020b; Reinert, 2020; Suicide Prevention Resource Centre [SPRC], 2020)   - Arrange coverage periods if possible, and let youth know when you will be away (SPRC, 2020) - Connect with youth and caregivers using institutional (not personal) devices (ASCA, n.d.) - Have a clear schedule for when you meet with youth (Reinert, 2020) |
| **3. Consent procedures** | - Consent for service form should include the name and contact information for multiple adult contacts, in case one is not available (Sayal et al., 2019)^a^   - Consent should detail who the youth/caregiver should contact in case of crisis, especially when school-based eMental health services are not available (Nelson et al., 2011)   - Consent for service form should also describe risks and benefits of eMental health (Nelson et al., 2011)   - Service providers should maintain communication with caregivers to ensure continuity of care (Nelson et al., 2011) | - Obtain caregiver consent/youth assent to conduct session remotely (Abrams, 2020; Alvord, Baker & Associates, LLC, n.d.; APA, 2020a; CYMH & SMH, 2020; NASP, 2020a)   - Consent topics include telling the youth you will not record the session without permission; whether you will use a webcam during the session (if relevant); using a secure internet connection; having a back-up plan (what to do if there are technology issues); having the name and contact information for at least one emergency contact; and knowing the closest emergency room to where the youth is (APA, 2020a) - The consent form can also detail (NASP, 2020a):   - A description of the eMental health service   - Any required technical considerations   - What you can and can’t do (i.e., eMental health limits)   - Expectations of service provider, youth and caregiver   - Emergency contacts and multiple communication options   - What will happen if the youth is determined to be in immediate safety risk and/or what will happen if caregivers cannot provide supervision   - Consent for youth to participate in eMental health |
| **4. Session logistics** | - Ask youth participants to provide contact information for themselves and one supportive adult at the beginning of each session in case you are disconnected and need to get support to them (Radovic et al., 2018) - Service providers require training and clinical supervision to provide suicide assessment via eHealth (Nielssen et al., 2015) - Have a back-up plan in case there are internet or technology issues *before* the session starts, and ensure the youth understands this plan (Nelson et al., 2011) - If immediate risk is identified through standard assessment, refer youth to crisis mental health services (Arjadi et al., 2018; Nielssen et al., 2015) - Understand relevant professional requirements for providing eMental health services to youth at risk for suicide within the school setting (Haas et al., 2008) | - Make sure you are competent with whatever virtual/remote platform you plan to use and in providing risk assessment virtually/remotely (Abrams, 2020; Alvord, Baker & Associates, LLC, n.d; CYMH & SMH, 2020) Service providers need training and supervision (ZERO Suicide Institute, n.d.c). Have a plan for receiving remote supervision as needed (NASP, 2020a).   - Check your technology right before the session (e.g., for software updates) (Alvord, Baker & Associates, LLC, n.d)   - Ensure technology meets relevant privacy requirements (ZERO Suicide Institute, n.d.a). Understand relevant local laws and regulations around providing eMental health services (Telligen Health Information Technology Regional Extension Centre & Great Plains Telehealth Resource & Assistance Centre, 2014).   - Plan a practice session with youth, if possible, to make sure you are both comfortable (ZERO Suicide Institute, n.d.a) - Make sure you can access your school division’s suicide risk assessment protocol electronically (NASP, 2020a)   - Completing a full suicide risk assessment virtually may be difficult, and so it is okay for service providers to focus on the most critical information needed to assess risk (SPRC, 2020) - Verify the youth’s identity at the start of the session (if can’t see them/haven’t met them before) (Alvord, Baker & Associates, LLC, n.d.) - Confirm consent/assent (Alvord, Baker & Associates, LLC, n.d.) - Review privacy (Alvord, Baker & Associates, LLC, n.d.; CYMH & SMH, 2020)   - Check if the youth is safe to talk (Perlman et al., 2011). Be prepared for next steps if they say no.   - Take steps to mitigate any potential privacy issues (ASCA, n.d.). For example, having the youth play white noise from an app (SPRC, 2020). Discuss what to do if privacy is interrupted, like if a sibling walks in (e.g., use of code word, hitting mute, switching to chat) (Reinert, 2020; ZERO Suicide Institute, n.d.b). - Review safety precautions (Alvord, Baker & Associates, LLC, n.d.)   - Confirm the youth’s physical location at start of the session (Alvord, Baker & Associates, LLC, n.d.; APA, 2020b; MHTTC Network, n.d.; NASP, 2020a; SPRC, 2020; Telligen Health Information Technology Regional Extension Centre & Great Plains Telehealth Resource & Assistance Centre, 2014; ZERO Suicide Institute, n.d.b)   - Have a back-up plan for what you will do if technology difficulties occur. Know how you can reach youth if you get disconnected (Alvord, Baker & Associates, LLC, n.d.; APA, 2020b; CYMH & SMH, 2020; MHTTC Network, n.d.; NASP, 2020a; ZERO Suicide Institute, n.d.a) and let them know how they can reach you (Alvord, Baker & Associates, LLC, n.d).   - Have a list of urgent and non-urgent nearby resources ready (Alvord, Baker & Associates, LLC, n.d). Know the 24/7 emergency services in your area and who you can/need to contact if the youth is at risk of suicide (NASP, 2020).   - Make sure you have up-to-date and accurate emergency contact information (that works) for at least one primary caregiver, and ensure this person is available in case they are needed (Alvord, Baker & Associates, LLC, n.d; APA, 2020a; Cox, 2020; MHTTC Network, n.d.; NASP, 2020a; SPRC, 2020; ZERO Suicide Institute, n.d.a; ZERO Suicide Institute, n.d.b). Can consider having information for one contact inside the house and one outside (Cox, 2020). - Monitor how youth is feeling (e.g., through messaging) throughout the session and slow things down if needed (Nassar, Costello, & Wolf-Prussan, 2020) - Have a plan for how you will stay connected to the youth if you need to contact emergency services (especially if you are connecting with them by phone) (SPRC, 2020; ZERO Suicide Institute, n.d.b). Stay connected with youth while you call 911 and until emergency services arrive (CYMH & SMH, 2020; MHTTC Network, n.d.). Maintain constant verbal (and if possible visual) contact until resources arrive (NASP, 2020). - Documentation is key – make sure to document when assessment started and ended, what platform you used/any technological difficulties, specific topics covered, and any other issues that occurred (CYMH & SMH, 2020; NASP, 2020a; ZERO Suicide Institute, n.d.a; ZERO Suicide Institute, n.d.b) - Close the session by asking what can be improved and making a plan for your next meeting (Cox, 2020; Zero Suicide Institute, n.d.b). If youth miss the session, check in with them to see what is going on and how you can adjust to make it easier/more comfortable for them to attend (CYMH & SMH, 2020). |
| **5. Safety planning** | - Online risk assessments follow same basic steps as in-person risk assessments (e.g., completing a safety plan; having information for in-person resources and emergency services ready before session, in case needed; Nielssen et al., 2015)^a^   - Like in-person assessments, specific suicide risk assessment protocols and procedures outlining steps and reporting requirements should be provided to all service providers (Nielssen et al., 2015; Sayal et al., 2019)   - Core professional principles and ethics remain critical in the eHealth environment (Nelson et al., 2011) - If risk is not immediate, develop a safety plan – send the safety plan to the youth and their caregiver, and include contact information for 24 hour resources (Nielssen et al., 2015; Fairchild et al., 2020)   - If possible, continually monitor risk via weekly online assessments; if risk increases, contact the caregiver and the youth (Nielssen et al., 2015) - Consider using ongoing screening data, and having youth provide data on an agreed upon schedule (e.g., daily, at agreed upon times, through weekly online assessments), to remotely monitor risk (Arjadi et al., 2018; Goodday et al., 2020; Nielssen et al., 2015)   - Providing youth with ongoing, personalized feedback on suicide risk indicators and then giving them the option to receive online counselling can have a positive impact on engagement in professional mental health treatment (Anderson et al., 2017; King et al., 2015)   - More check-ins may be required than when youth are in school, depending on the level of isolation of the youth (Nelson et al., 2011) - Inform caregivers if suicide risk issues arise, and provide clear guidelines to caregivers on how to manage risk and seek appropriate help (Anderson et al., 2017)^a^   - Notify caregivers of the risk, recommended next steps, and a list of appropriate crisis services and support agencies (Anderson et al., 2017)   - Consider having a caregiver sit with the youth when conducting risk assessment, if safe and age-appropriate (Fairchild et al., 2020; Thomas et al., 2018) | - Overall, safety planning is the same as in person (Telligen Health Information Technology Regional Extension Centre & Great Plains Telehealth Resource & Assistance Centre, 2014). Work together to build a safety plan (e.g., by sharing screen) (ZERO Suicide Institute, n.d.a), and find a way to get the plan to them (e.g., email). (SPRC, 2020; ZERO Suicide Institute, n.d.a; ZERO Suicide Institute, n.d.b; ZERO Suicide Institute, n.d.c)   - Ask about increased access to lethal means (e.g., medication, firearms) (SPRC, 2020)   - Ask about additional COVID-19 related risk factors (e.g., social isolation, family financial stress) (SPRC, 2020) - Consider using virtual safety planning tools, like the My3 app (NASP, 2020a) - Check-ins may need to happen more often (SPRC, 2020; ZERO Suicide Institute, n.d.c). Consider using a short screener during check-ins to remotely monitor risk (NIMH, 2020; SPRC, 2020). Youth could also use an app to rate their mood/suicidal ideation daily, so they know when they might need urgent care (Kaslow, 2014). Can also use check-ins to review and update safety plans (Van Dyk et al., 2020). - Figure out a way for the youth to get a copy of their safety plan (e.g., text it to them, have them take a screenshot) (SPRC, 2020; ZERO Suicide Institute, n.d.a; ZERO Suicide Institute, n.d.b; ZERO Suicide Institute, n.d.c)   - Let the primary caregiver know you have developed a safety plan (CYMH & SMH, 2020; NASP, 2020a)   - Develop a plan with caregivers on how they can access support for themselves (ASCA, n.d.) - Identify coping strategies on the safety plan that can be done during COVID-19 (e.g., virtual activities, virtual connection with friends) (SPRC, 2020) - Encourage the youth to keep a daily schedule and make plans for each day (SPRC, 2020) |
| **6. Internet privacy** | - Send virtual session invitations via a secure and encrypted email (Thomas et al., 2018) - Give each youth a unique, non-identifying username and password (Arjadi et al., 2018; Haas et al., 2008) - Store youth information (e.g., email addresses) in an encrypted computer system (Haas et al., 2008) and use encrypted point-to-point technologies when videoconferencing (Nelson et al., 2011) - Ensure virtual session hosting platform is compliant with relevant health privacy law in your area (e.g., HIPAA) (Thomas et al., 2018)   - Determine who is in the room on both sides (other than the youth and service provider), and ensure that the people in the rooms meet privacy law requirements (Nelson et al., 2011) | - No specific recommendations provided |

**Appendix 2 References**

[Abrams Z. *Ethical guidance for the COVID-19 era*. American Psychological Association. June 2020. https://www.apa.org/monitor/2020/06/ce-corner-ethical-guidance](Abrams%20Z.%20Ethical%20guidance%20for%20the%20COVID-19%20era.%20American%20Psychological%20Association.%20June%202020.%20https://www.apa.org/monitor/2020/06/ce-corner-ethical-guidance)

[Alvord, Baker & Associates, LLC. *TeleMental health via video conferencing checklist*. National Register. n.d. https://www.nationalregister.org/wp-content/uploads/2020/03/for-3-18-20-post-Alvord-Telehealth-Checklist.pdf](Alvord,%20Baker%20&%20Associates,%20LLC.%20TeleMental%20health%20via%20video%20conferencing%20checklist.%20National%20Register.%20n.d.%20https://www.nationalregister.org/wp-content/uploads/2020/03/for-3-18-20-post-Alvord-Telehealth-Checklist.pdf)

[American Psychiatric Association. *How to prepare for a video appointment with your mental health clinician.* SMI Adviser. 2020. https://smiadviser.org/wp-content/uploads/2020/04/How-to-Prepare-for-a-Video-Appointment.pdf](American%20Psychiatric%20Association.%20How%20to%20prepare%20for%20a%20video%20appointment%20with%20your%20mental%20health%20clinician.%20SMI%20Adviser.%202020.%20https://smiadviser.org/wp-content/uploads/2020/04/How-to-Prepare-for-a-Video-Appointment.pdf)

[American Psychological Association [APA]. *Informed consent checklist for telepsychological services.* March 2020a. https://www.apa.org/practice/programs/dmhi/research-information/informed-consent-checklist](American%20Psychological%20Association%20%5bAPA%5d.%20Informed%20consent%20checklist%20for%20telepsychological%20services.%20March%202020a.%20https://www.apa.org/practice/programs/dmhi/research-information/informed-consent-checklist)

[American Psychological Association [APA]. *Office and telepsychology checklist for telepsychological services.* March 2020b. https://www.apa.org/practice/programs/dmhi/research-information/telepsychological-services-checklist](American%20Psychological%20Association%20%5bAPA%5d.%20Office%20and%20telepsychology%20checklist%20for%20telepsychological%20services.%20March%202020b.%20https://www.apa.org/practice/programs/dmhi/research-information/telepsychological-services-checklist)

[American School Counselor Association [ASCA]. *Planning for virtual/distance school counselling during an emergency shutdown*. n.d. https://www.schoolcounselor.org/asca/media/asca/home/EmergencyShutdown.pdf](American%20School%20Counselor%20Association%20%5bASCA%5d.%20Planning%20for%20virtual/distance%20school%20counselling%20during%20an%20emergency%20shutdown.%20n.d.%20https://www.schoolcounselor.org/asca/media/asca/home/EmergencyShutdown.pdf)

[Anderson RA, Rees CS, Finlay-Jones AL. Internet-based cognitive-behavioural therapy for young people with obsessive-compulsive disorder: Lessons learned. J*ournal of Obsessive-Compulsive and Related Disorders*. 2017;15:7-12. https://doi.org/10.1016/j.jocrd.2017.08.001](Anderson%20RA,%20Rees%20CS,%20Finlay-Jones%20AL.%20Internet-based%20cognitive-behavioural%20therapy%20for%20young%20people%20with%20obsessive-compulsive%20disorder:%20Lessons%20learned. Journal%20of%20Obsessive-Compulsive%20and%20Related%20Disorders. 2017;15:7-12.%20https://doi.org/10.1016/j.jocrd.2017.08.001)

[Arjadi R, Nauta MH, Scholte WF, Hollon SD, Chowdhary N, Suryani AO, et al. Internet-based behavioural activation with lay counsellor support versus online minimal psychoeducation without support for treatment of depression: A randomised controlled trial in Indonesia. *The Lancet Psychiatry*. 2018;5(9):707-716. https://doi.org/10.1016/S2215-0366(18)30223-2](Arjadi%20R,%20Nauta%20MH,%20Scholte%20WF,%20Hollon%20SD,%20Chowdhary%20N,%20Suryani%20AO,%20et%20al.%20Internet-based%20behavioural%20activation%20with%20lay%20counsellor%20support%20versus%20online%20minimal%20psychoeducation%20without%20support%20for%20treatment%20of%20depression:%20A%20randomised%20controlled%20trial%20in%20Indonesia. The%20Lancet%20Psychiatry. 2018;5(9):707-716.%20https://doi.org/10.1016/S2215-0366(18)30223-2)

[Cox J.](Cox J. Telemental health 101 [Video]. National Center for School Mental Health. March 2020. https://www.youtube.com/watch?v=3A74lXLTABw) *[Telemental health 101 [Video]](Cox J. Telemental health 101 [Video]. National Center for School Mental Health. March 2020. https://www.youtube.com/watch?v=3A74lXLTABw)*[. National Center for School Mental Health. March 2020. https://www.youtube.com/watch?v=3A74lXLTABw](Cox J. Telemental health 101 [Video]. National Center for School Mental Health. March 2020. https://www.youtube.com/watch?v=3A74lXLTABw)

[Fairchild RM, Ferng-Kuo SF, Rahmouni H, Hardesty D. Telehealth increases access to care for children dealing with suicidality, depression, and anxiety in rural emergency departments. *Telemedicine and e-Health*. 2020. https://doi.org/10.1089/tmj.2019.0253](Fairchild%20RM,%20Ferng-Kuo%20SF,%20Rahmouni%20H,%20Hardesty%20D.%20%20Telehealth%20increases%20access%20to%20care%20for%20children%20dealing%20with%20suicidality,%20depression,%20and%20anxiety%20in%20rural%20emergency%20departments. Telemedicine%20and%20e-Health.%202020.%20https://doi.org/10.1089/tmj.2019.0253)

[Goodday SM, Atkinson L, Goodwin G, Saunders K, South M, Mackay C, et al. The true colours remote symptom monitoring system: A decade of evolution. *Journal of Medical Internet Research*. 2020;22(1):e15188. https://doi.org/10.2196/15188](Goodday%20SM,%20Atkinson%20L,%20Goodwin%20G,%20Saunders%20K,%20South%20M,%20Mackay%20C,%20et%20al.%20The%20true%20colours%20remote%20symptom%20monitoring%20system:%20A%20decade%20of%20evolution. Journal%20of%20Medical%20Internet%20Research. 2020;22(1):e15188.%20https://doi.org/10.2196/15188)

[Haas A, Koestner B, Rosenberg J, Moore D, Garlow SJ, Sedway J, et al. An interactive web-based method of outreach to college students at risk for suicide. *Journal of American College Health*. 2008;57(1):15-22. https://doi.org/10.3200/JACH.57.1.15-22](Haas%20A,%20Koestner%20B,%20Rosenberg%20J,%20Moore%20D,%20Garlow%20SJ,%20Sedway%20J,%20et%20al.%20An%20interactive%20web-based%20method%20of%20outreach%20to%20college%20students%20at%20risk%20for%20suicide. Journal%20of%20American%20College%20Health. 2008;57(1):15-22.%20https://doi.org/10.3200/JACH.57.1.15-22)

[Kaslow NJ. *Preventing suicide (14) [Podcast episode]*. In Speaking of psychology. American Psychological Association. 2014. https://www.apa.org/research/action/speaking-of-psychology/preventing-suicide](Kaslow%20NJ.%20%20Preventing%20suicide%20(14)%20%5bPodcast%20episode%5d.%20In%20Speaking%20of%20psychology.%20American%20Psychological%20Association.%202014.%20https://www.apa.org/research/action/speaking-of-psychology/preventing-suicide)

[King CA, Eisenberg D, Zheng K, Czyz E, Kramer A, Horwitz A, Chermack S. Online suicide risk screening and intervention with college students: A pilot randomized controlled trial. *Journal of Consulting and Clinical Psychology*. 2015;83(3): 630-636. https://doi.org/10.1037/a0038805](King%20CA,%20Eisenberg%20D,%20Zheng%20K,%20Czyz%20E,%20Kramer%20A,%20Horwitz%20A,%20Chermack%20S.%20Online%20suicide%20risk%20screening%20and%20intervention%20with%20college%20students:%20A%20pilot%20randomized%20controlled%20trial. Journal%20of%20Consulting%20and%20Clinical%20Psychology. 2015;83(3):%20630-636.%20https://doi.org/10.1037/a0038805)

[Lowenstein L. *Creative interventions for online therapy with children: Techniques to build rapport*. Liana Lowenstein. n.d. http://www.lianalowenstein.com/artcileProfTeleplayLowenstein.pdf](Lowenstein%20L.%20Creative%20interventions%20for%20online%20therapy%20with%20children:%20Techniques%20to%20build%20rapport.%20Liana%20Lowenstein.%20n.d.%20http://www.lianalowenstein.com/artcileProfTeleplayLowenstein.pdf)

[Mental Health Technology Transfer Center [MHTTC] Network, Northeast and Caribbean (HHS Region 2). *Engaging with clients over the telephone and using texts*. Mental Health Technology Transfer Center Network. n.d. https://mhttcnetwork.org/sites/default/files/2020-05/Engaging%20with%20Clients%20Over%20the%20Telephone%20and%20Using%20Texts_FINALpdf.pdf](Mental%20Health%20Technology%20Transfer%20Center%20%5bMHTTC%5d%20Network,%20Northeast%20and%20Caribbean%20(HHS%20Region%202).%20Engaging%20with%20clients%20over%20the%20telephone%20and%20using%20texts.%20Mental%20Health%20Technology%20Transfer%20Center%20Network.%20n.d.%20https://mhttcnetwork.org/sites/default/files/2020-05/Engaging%20with%20Clients%20Over%20the%20Telephone%20and%20Using%20Texts_FINALpdf.pdf)

[Nassar C, Costello JK, Wolf-Prusan L. *Telehealth learning and consultation (TLC) Tuesdays, April 28: Telehealth troubleshooting [webinar slides].* Mental Health Technology Transfer Center Network. April 20202. https://mhttcnetwork.org/centers/mid-america-mhttc/telehealth-learning-and-consultation-tlc-tuesdays](Nassar%20C,%20Costello%20JK,%20Wolf-Prusan%20L.%20Telehealth%20learning%20and%20consultation%20(TLC)%20Tuesdays,%20April%2028:%20Telehealth%20troubleshooting%20%5bwebinar%20slides%5d.%20Mental%20Health%20Technology%20Transfer%20Center%20Network.%20April%2020202.%20https://mhttcnetwork.org/centers/mid-america-mhttc/telehealth-learning-and-consultation-tlc-tuesdays)

[National Association of School Psychologists [NASP]. *Comprehensive suicide prevention in a time of distance learning (handout)*. NASP Online. 2020a. https://www.nasponline.org/resources-and-publications/resources-and-podcasts/covid-19-resource-center/crisis-and-mental-health-resources/comprehensive-school-suicide-prevention-in-a-time-of-distance-learning](National%20Association%20of%20School%20Psychologists%20%5bNASP%5d.%20Comprehensive%20suicide%20prevention%20in%20a%20time%20of%20distance%20learning%20(handout).%20NASP%20Online.%202020a.%20https://www.nasponline.org/resources-and-publications/resources-and-podcasts/covid-19-resource-center/crisis-and-mental-health-resources/comprehensive-school-suicide-prevention-in-a-time-of-distance-learning)

[National Association of School Psychologists [NASP]. *Virtual service delivery in response to COVID-19 disruptions.* NASP Online. 2020b. https://www.nasponline.org/resources-and-publications/resources-and-podcasts/school-climate-safety-and-crisis/health-crisis-resources/virtual-service-delivery-in-response-to-covid-19-disruptions](National%20Association%20of%20School%20Psychologists%20%5bNASP%5d.%20Virtual%20service%20delivery%20in%20response%20to%20COVID-19%20disruptions.%20NASP%20Online.%202020b.%20https://www.nasponline.org/resources-and-publications/resources-and-podcasts/school-climate-safety-and-crisis/health-crisis-resources/virtual-service-delivery-in-response-to-covid-19-disruptions)

[National Institute of Mental Health [NIMH]. C*OVID-19: Youth suicide risk screening pathway [Infographic]*. NIMH. May 2020. https://www.nimh.nih.gov/research/research-conducted-at-nimh/asq-toolkit-materials/inpatient/pdfs/covid-19_youth_suicide_risk_screening_pathway_160183.pdf](National%20Institute%20of%20Mental%20Health%20%5bNIMH%5d.%20COVID-19:%20Youth%20suicide%20risk%20screening%20pathway%20%5bInfographic%5d.%20NIMH.%20May%202020.%20https://www.nimh.nih.gov/research/research-conducted-at-nimh/asq-toolkit-materials/inpatient/pdfs/covid-19_youth_suicide_risk_screening_pathway_160183.pdf)

[Navarro P, Sheffield J, Edirippulige S, Bambling M. Exploring mental health professionals’ perspectives of text-based online counseling effectiveness with young people: Mixed methods pilot study. *JMIR Mental Health*. 2020;7(1):e15564. https://doi.org/10.2196/15564](Navarro%20P,%20Sheffield%20J,%20Edirippulige%20S,%20Bambling%20M.%20Exploring%20mental%20health%20professionals’%20perspectives%20of%20text-based%20online%20counseling%20effectiveness%20with%20young%20people:%20Mixed%20methods%20pilot%20study. JMIR%20Mental%20Health. 2020;7(1):e15564.%20https://doi.org/10.2196/15564)

[Nelson EL, Bui TN, Velasquez SE. Telepsychology: Research and practice overview. *Child and Adolescent Psychiatric Clinics*. 2011;20(1):67-79. https://doi.org/10.1016/j.chc.2010.08.005](Nelson%20EL,%20Bui%20TN,%20Velasquez%20SE.%20Telepsychology:%20Research%20and%20practice%20overview. Child%20and%20Adolescent%20Psychiatric%20Clinics. 2011;20(1):67-79.%20https://doi.org/10.1016/j.chc.2010.08.005)

[Nielssen O, Dear BF, Staples LG, Dear R, Ryan K, Purtell C, Titov N. Procedures for risk management and a review of crisis referrals from the MindSpot Clinic, a national service for the remote assessment and treatment of anxiety and depression. *BMC Psychiatry*. 2015;15(1):1-6. https://doi.org/10.1186/s12888-015-0676-6](Nielssen%20O,%20Dear%20BF,%20Staples%20LG,%20Dear%20R,%20Ryan%20K,%20Purtell%20C,%20Titov%20N.%20%20Procedures%20for%20risk%20management%20and%20a%20review%20of%20crisis%20referrals%20from%20the%20MindSpot%20Clinic,%20a%20national%20service%20for%20the%20remote%20assessment%20and%20treatment%20of%20anxiety%20and%20depression. BMC%20Psychiatry. 2015;15(1):1-6.%20https://doi.org/10.1186/s12888-015-0676-6)

[Ontario Centre of Excellence for Child, & Youth Mental Health [CYMH] and School Mental Health Ontario [SMH]. *Virtual care 101 webinar: Questions and answers*. 2020. https://www.cymh.ca/en/projects/resources/covid-19/covid19virtualcarewebinarQandAwcag.pdf](Ontario%20Centre%20of%20Excellence%20for%20Child,%20&%20Youth%20Mental%20Health%20%5bCYMH%5d%20and%20School%20Mental%20Health%20Ontario%20%5bSMH%5d.%20Virtual%20care%20101%20webinar:%20Questions%20and%20answers.%202020.%20https://www.cymh.ca/en/projects/resources/covid-19/covid19virtualcarewebinarQandAwcag.pdf)

[Perlman CM, Neufeld E, Martin L, Goy M, Hirdes JP. *Suicide risk assessment inventory: A resource guide for Canadian health care organizations.* Patient Safety Institute. 2011. https://www.patientsafetyinstitute.ca/en/toolsResources/SuicideRisk/Documents/Suicide%20Risk%20Assessment%20Guide.pdf](Perlman%20CM,%20Neufeld%20E,%20Martin%20L,%20Goy%20M,%20Hirdes%20JP.%20%20Suicide%20risk%20assessment%20inventory:%20A%20resource%20guide%20for%20Canadian%20health%20care%20organizations.%20Patient%20Safety%20Institute.%202011.%20https://www.patientsafetyinstitute.ca/en/toolsResources/SuicideRisk/Documents/Suicide%20Risk%20Assessment%20Guide.pdf)

[Radovic A, Gmelin T, Hua J, Long C, Stein BD, Miller E. Supporting Our Valued Adolescents (SOVA), a social media website for adolescents with depression and/or anxiety: Technological feasibility, usability, and acceptability study. *JMIR Mental Health*. 2018;5(1):e17. https://doi.org/10.2196/mental.9441](Radovic%20A,%20Gmelin%20T,%20Hua%20J,%20Long%20C,%20Stein%20BD,%20Miller%20E.%20Supporting%20Our%20Valued%20Adolescents%20(SOVA),%20a%20social%20media%20website%20for%20adolescents%20with%20depression%20and/or%20anxiety:%20Technological%20feasibility,%20usability,%20and%20acceptability%20study. JMIR%20Mental%20Health. 2018;5(1):e17.%20https://doi.org/10.2196/mental.9441)

[Reinert P. *Telehealth guidelines for school mental health professionals: Strategies for engaging students and building resilience.* School Mental Health. May 2020. http://www.schoolmentalhealth.org/media/SOM/Microsites/NCSMH/Documents/COVID-19/Telehealth-for-Mental-Health-DRAFT-7.pdf](Reinert%20P.%20%20Telehealth%20guidelines%20for%20school%20mental%20health%20professionals:%20Strategies%20for%20engaging%20students%20and%20building%20resilience.%20School%20Mental%20Health.%20May%202020.%20http://www.schoolmentalhealth.org/media/SOM/Microsites/NCSMH/Documents/COVID-19/Telehealth-for-Mental-Health-DRAFT-7.pdf)

[Sayal K, Roe J, Ball H, Atha C, Kaylor-Hughes C, Guo B, Townsend E, Morriss R. Feasibility of a randomised controlled trial of remotely delivered problem-solving cognitive behaviour therapy versus usual care for young people with depression and repeat self-harm: lessons learnt (e-DASH). *BMC Psychiatry*. 2019;19(42):1-12. https://doi.org/10.1186/s12888-018-2005-3](Sayal%20K,%20Roe%20J,%20Ball%20H,%20Atha%20C,%20Kaylor-Hughes%20C,%20Guo%20B,%20Townsend%20E,%20Morriss%20R.%20%20Feasibility%20of%20a%20randomised%20controlled%20trial%20of%20remotely%20delivered%20problem-solving%20cognitive%20behaviour%20therapy%20versus%20usual%20care%20for%20young%20people%20with%20depression%20and%20repeat%20self-harm:%20lessons%20learnt%20(e-DASH). BMC%20Psychiatry. 2019;19(42):1-12.%20https://doi.org/10.1186/s12888-018-2005-3%20)

[Suicide Prevention Resource Center [SPRC]. *Treating suicidal patients during COVID-19: Best practices and telehealth.* April 2020. https://www.sprc.org/events-trainings/treating-suicidal-patients-during-covid-19-best-practices-telehealth](Suicide%20Prevention%20Resource%20Center%20%5bSPRC%5d.%20Treating%20suicidal%20patients%20during%20COVID-19:%20Best%20practices%20and%20telehealth.%20April%202020.%20https://www.sprc.org/events-trainings/treating-suicidal-patients-during-covid-19-best-practices-telehealth)

[Telligen Health Information Technology Regional Extension Center [THITREC] and Great Plains Telehealth Resource & Assistance Center [GPTRAC]. *Telehealth start-up and resource guide. Version 1.1*. 2014. https://www.ruralcenter.org/resource-library/telehealth-start-up-and-resource-guide](Telligen%20Health%20Information%20Technology%20Regional%20Extension%20Center%20%5bTHITREC%5d%20and%20Great%20Plains%20Telehealth%20Resource%20&%20Assistance%20Center%20%5bGPTRAC%5d.%20Telehealth%20start-up%20and%20resource%20guide.%20Version%201.1.%202014.%20https://www.ruralcenter.org/resource-library/telehealth-start-up-and-resource-guide)

Thomas JF, Novins DK, Hosokawa PW. The use of telepsychiatry to provide cost-efficient care during pediatric mental health emergencies. *Psychiatric Services*. 2018;69(2):161-168. https://doi.org/10.1176/appi.ps.201700140.

[van Dyk IS, Kroll JL, Martinez RG, Emerson ND, Bursch B. *COVID-19 tips: Building rapport with youth via telehealth*. 2020. https://www.williamjames.edu/community/resource-hub/upload/TelehealthTipSheet-COVIDChildVersionFINAL.pdf.](van%20Dyk%20IS,%20Kroll%20JL,%20Martinez%20RG,%20Emerson%20ND,%20Bursch%20B.%20%20COVID-19%20tips:%20Building%20rapport%20with%20youth%20via%20telehealth.%202020.%20https://www.williamjames.edu/community/resource-hub/upload/TelehealthTipSheet-COVIDChildVersionFINAL.pdf.%20)

[ZEROSuicide Institute. *Telehealth and suicide care during the COVID-19 pandemic.* n.d.a. http://zerosuicide.edc.org/sites/default/files/Telehealth%20and%20Suicide%20Care%20During%20the%20COVID-19%20Pandemic.pdf](ZEROSuicide%20Institute.%20Telehealth%20and%20suicide%20care%20during%20the%20COVID-19%20pandemic.%20n.d.a.%20http://zerosuicide.edc.org/sites/default/files/Telehealth%20and%20Suicide%20Care%20During%20the%20COVID-19%20Pandemic.pdf)

[ZEROSuicide Institute. *Telehealth tips: Managing suicidal clients during the COVID-19 pandemic. n.d.b.* http://zerosuicide.edc.org/sites/default/files/Telehealth%20Tips%20with%20Suicidal%20Clients%20-%20FINAL.pdf](ZEROSuicide%20Institute.%20Telehealth%20tips:%20Managing%20suicidal%20clients%20during%20the%20COVID-19%20pandemic.%20n.d.b.%20http://zerosuicide.edc.org/sites/default/files/Telehealth%20Tips%20with%20Suicidal%20Clients%20-%20FINAL.pdf)

[ZEROSuicide Institute. *Zero suicide implementation during COVID-19 response.* n.d.c. http://zerosuicide.edc.org/sites/default/files/Zero%20Suicide%20Implementation%20During%20the%20COVID-19%20Response.pdf](ZEROSuicide%20Institute.%20Zero%20suicide%20implementation%20during%20COVID-19%20response.%20n.d.c.%20http://zerosuicide.edc.org/sites/default/files/Zero%20Suicide%20Implementation%20During%20the%20COVID-19%20Response.pdf)
